# Mycobacterial-specific secretion of cytokines and chemokines in healthcare workers with apparent resistance to infection with *Mycobacterium tuberculosis*

**DOI:** 10.3389/fimmu.2023.1176615

**Published:** 2023-05-18

**Authors:** Muki Shehu Shey, Avuyonke Balfour, Nomawethu Masina, Abulele Bekiswa, Charlotte Schutz, Rene Goliath, Rachel Dielle, Patrick DMC. Katoto, Katalin Andrea Wilkinson, David Lewinsohn, Deborah Anne Lewinsohn, Graeme Meintjes

**Affiliations:** ^1^ Department of Medicine, Faculty of Health Sciences, University of Cape Town, Cape Town, South Africa; ^2^ Wellcome Centre for Infectious Disease Research in Africa (CIDRI-Africa), Faculty of Health Sciences, University of Cape Town, Cape Town, South Africa; ^3^ Institute of Infectious Disease and Molecular Medicine (IDM), Faculty of Health Sciences, University of Cape Town, Cape Town, South Africa; ^4^ Cochrane South Africa, South African Medical Research Council, Cape Town, South Africa; ^5^ Centre for General Medicine and Global Health, Department of Medicine, University of Cape Town, Cape Town, South Africa; ^6^ Centre for Tropical Diseases and Global Health and Department of Internal Medicine , Catholic University of Bukavu, Bukavu, Democratic Republic of Congo; ^7^ Tuberculosis Laboratory, The Francis Crick Institute, London, United Kingdom; ^8^ Division of Pulmonary and Critical Care Medicine, Department of Medicine, Oregon Health and Science University, Portland, OR, United States; ^9^ Division of Infectious Diseases, Department of Paediatrics, Oregon Health and Science University, Portland, OR, United States

**Keywords:** latent TB infection (LTBI), resister, cytokines, chemokines, *Mycobacterium tuberculosis*, antibodies

## Abstract

**Background:**

Currently, diagnosis of latent TB infection (LTBI) is based on the secretion of IFN-γ in response to *Mycobacterium tuberculosis* (Mtb) antigens, the absence of which is regarded as no infection. Some individuals appear to resist Mtb infection despite sustained exposure (resisters). In this study, we aimed to assess cytokines, chemokines and antibodies that may be associated with resistance to Mtb infection. We hypothesized that there may be an alternative immune response to Mtb exposure in the absence of IFN-γ in resisters.

**Methods:**

We enrolled HIV-uninfected healthcare workers who had worked in high TB-exposure environments for 5 years or longer. We screened them for LTBI using the tuberculin skin test and the QuantiFERON-TB Gold Plus assay. We performed multiplex Luminex to measure concentrations of T cell-associated cytokines and chemokines as well as total antibodies in plasma collected from unstimulated fresh whole blood and supernatants from QuantiFERON-TB Gold Plus tubes following incubation of whole blood for 16-24 hours with ESAT6/CFP10 peptides.

**Results:**

Samples from 78 individuals were analyzed: 33 resisters (TST<10mm; IGRA<0.35 IU/mL), 33 with LTBI (TST≥10mm and IGRA≥0.35 IU/mL) and 12 discordant (TST=0mm; IGRA≥1.0 IU/mL). There were no differences in concentrations of cytokines and chemokines in plasma between the different groups. Resisters had significantly lower concentrations of IFN-γ, IL-2, TNF-α, MIP-1α, MIP-1β, ITAC, IL-13 and GM-CSF in supernatants compared with LTBI group. There were no significant differences in the concentrations in supernatants of IL-10, IL-1β, IL-17A, IL-21, IL-23, MIP-3α, IL-4, IL-5, IL-6, IL-7, IL-8, Fractalkine and IL-12p70 between the groups. We observed that resisters had similar concentrations of total antibodies (IgG1, IgG2, IgG3, IgG4, IgA, and IgM) in plasma and supernatants compared to the LTBI and discordant groups.

**Conclusion:**

Resistance to Mtb infection despite sustained exposure is associated with lower Mtb-specific secretion of Th1-associated cytokines and chemokines. However, resisters showed secreted concentrations after Mtb stimulation of total antibodies and cytokines/chemokines associated with innate and Th17 immune responses similar to those with Mtb infection. This suggests an ability to mount non-IFN-γ immune responses to Mtb in apparent resisters.

## 1 Introduction

*Mycobacterium tuberculosis* (Mtb) is the aetiologic agent for tuberculosis (TB). Exposure to Mtb can result in either infection or possible clearance. Infection can either be contained in the latent form known as latent TB infection (LTBI) or progress to active disease (1). Diagnosis of LTBI relies on interferon-gamma (IFN-γ) release in response to Mtb antigens as measured by IFN-γ release assays (IGRAs) and or delayed type hypersensitivity reactions to the purified protein derivative (PPD) administered as tuberculin skin test (TST) or Mantoux test. Despite these tests being used as gold standards for defining Mtb infection, there are limitations to these tests including over-reliance on IFN-γ (IGRA) and reactivity to protein antigens (TST) which might be non-specific for Mtb. Individuals may be exposed and/or infected with Mtb but generate a T cell immune response that is independent of IFN-γ (2). Most people living in communities where TB is endemic develop LTBI as defined by positive IGRA or TST. Individuals with LTBI have a 5-10% lifetime risk of developing TB disease, most of whom progress to TB within 1-2 years following conversion from a negative to positive TST or IGRA. However, despite sustained exposure, some individuals may either clear Mtb through an innate response without sensitization of the adaptive immune response or may develop an alternative T cell response not measured by IFN-γ. These individuals who remain IGRA and TST negative despite sustained exposures are termed “resisters” (reviewed in (3)).

Resisters exhibit low reactivity to TST and no IFN-γ release in response to Mtb antigens. Several studies have provided evidence of the existence of this resister phenotype (1, 4, 5). Household contacts (HHCs) studies in Uganda (5), and a recent multi-country study of HHCs of multidrug-resistant TB (MDR-TB) (4), showed that the resister phenotype constituted between 8- 11% of individuals exposed to Mtb. In a study of South African gold miners in service for over 15 years, 13% of the miners exhibited the resister phenotype (6). Factors responsible for this resister phenotype are not completely understood and may be related to either innate and/or alternative adaptive immune responses, genetic associations, epidemiological risk factors or a combination. Understanding the factors that contribute to this resister phenotype may be useful in developing the next generation of TB vaccines.

As resistance to Mtb infection or absence of infection despite known exposure is currently defined by the absence of IFN-γ measured by IGRA test in response to Mtb-derived antigens and or low-to-no reactivity to PPD in the TST, we sought to investigate the immune markers that are secreted in response to Mtb antigens in resisters as compared with individuals with LTBI and those with discordant results. We hypothesized that there may be alternative immune responses to Mtb exposure in the absence of IFN-γ in those considered resisters.

## 2 Methods

### 2.1 Participants

Participants were staff recruited at Brooklyn Chest Hospital, DP Marais Hospitals and Site B Ubuntu Clinic in Khayelitsha, Cape Town, South Africa. Participants in this study were healthcare workers and allied personnel working in TB treatment and care facilities with regular exposure to TB patients for 5 years or more; 18 years or older, HIV-uninfected, with no signs or symptoms of tuberculosis, no asthma or COPD and no previous TB episodes.

### 2.2 Screening for Mtb infection and enrollment

Participants completed a questionnaire with the help of the study staff to determine their eligibility, exclude signs and symptoms of active tuberculosis, and collect data on risk factors for Mtb exposure/infection including smoking history, frequency of contacts with patients with confirmed or suspected TB, comorbidities (diabetes, self-reported) and cumulative period working in a high TB exposure environment. Participants with no signs/symptoms of active TB, asthma or COPD were consented and tested for HIV infection using the rapid test (Abbot). If HIV-uninfected, they were eligible to continue the screening where 4mL of blood was taken into sodium heparin tubes for IGRA using the QuantiFERON-TB Gold Plus test. The blood was transported to the laboratory within 4 hours from collection, mixed and 1mL of blood was incubated for 16-24 hours in tubes containing TB antigens TB1 (antigens to stimulate mainly CD4 T cells), TB2 (antigens to stimulate both CD4 and CD8 T cells), as specified by the manufacturer (7), and tubes with mitogen and no antigens were used as positive and negative controls, respectively. After incubation, the tubes were centrifuged at 2000 x g for 15minutes, supernatants were collected from each tube and stored at -80 degrees Celsius. These supernatants were analyzed in batches using ELISA assays to determine if there was reactivity to the TB antigens assessed by the production of IFN-γ. IFN-γ levels of 0.35IU/mL or more were considered as positive while levels below 0.35IU/mL were considered as negative.

After collection of blood for IGRA, the PPD (for TST) was administered into the forearm by the study nurse. Induration from the TST was read after 48-72 hours. Induration of 10mm or more was considered positive and induration of less than 10mm was considered negative. Results from both the IGRA test and TST were then combined to determine those who were resistant to TB infection (resisters) and those with latent TB infection. The resister phenotype was defined as IFN-γ response to both TB1 and TB2 antigens less than 0.35IU/mL and TST induration less than 10mm. LTBI was defined as IFN-γ response to TB1 greater than 0.35IU/mL and TST induration greater than 10mm. These participants were then enrolled into the study. Some participants with discordant results (TST=0mm; IGRA≥1.0 IU/mL to TB1 antigens) were also enrolled to investigate whether cytokines, chemokines and total antibody responses in the absence of a TST induration with a high IGRA response were more similar to resisters or LTBI.

An additional 2mL of fresh blood was collected for plasma separation by centrifugation at 1500 x g for 15 minutes. Plasma was collected and stored at -80 degrees Celsius and later used for evaluation of soluble markers as described below.

### 2.3 Luminex assay

After classifying the individuals in the resister, LTBI and discordant groups as described above, we performed Luminex assay using both plasma and QuantiFERON-TB Gold Plus supernatants from the same participants to evaluate the concentrations of cytokines and chemokines associated with T cell function as well as innate immunity. We used a commercially available high sensitivity T cell panel kit from Merck-Millipore containing the following cytokines and chemokines: IL-2, TNF-α, MIP-1α, MIP-1β, ITAC, IL-13, GM-CSF, IL-10, IL-1β, IL-17A, IL-21, IL-23, MIP-3α, IL-4, IL-5, IL-6, IL-7, IL-8, Fractalkine and IL-12p70. We also assessed by Luminex the concentrations of total antibody isotypes in the same samples (both plasma and QuantiFERON-TB Gold Plus supernatants) using a commercially available isotyping panel (IgA, IgM, IgG1, IgG2, IgG3, IgG4) from Bio-Rad. Supernatants and plasma were diluted 2-fold for T cell cytokines/chemokines and 10 000-fold for antibodies and assays were performed according to manufacturers’ instructions (8, 9). For these analyses, we performed the Luminex experiments using samples from the TB1-stimulated tubes only as responses were more similar between the TB1 and TB2 tubes (**Supplementary Figure 1**). Data presented for IGRA results and Luminex from stimulated tubes are background-subtracted.

### 2.4 Statistical analyses

The concentrations of cytokines/chemokines and antibodies were categorized and analyzed statistically using R (version 4.2.1), STATA (version 15.1) and GraphPad Prism (version 9.2). The non-parametric Mann-Whitney test was used to compare groups (Resisters vs. LTBI or Resisters vs. Discordant and LTBI vs. Discordant) and the Bonferroni test was used to account for multiple comparisons. For marker investigation and comparison, dimensionality-reduction using Principal Component Analysis (PCA) in R was utilized (LTBI with resisters and all markers examined). Luminex cytokine and chemokine data were scaled and centered to conduct unsupervised PCA using the FactoMineR package (Version 2.7). As diagnostic indicators for latent TB infection, the diagnostic utility of selected cytokines assessed following stimulation with Mtb-specific antigens was evaluated using Receiver Operating Characteristic Curve (ROC) and Area Under the Curve (AUC) analyses. Spearman’s rank correlation was used to assess the strength and direction of the associations between IFN-response to TB1 and TB2 antigens measured by ELISA, and between IFN-measured by ELISA in the IGRA test (TB1) and IFN-measured by Luminex. P value ≤ 0.05 was considered as statistically significant.

## 3 Results

### 3.1 Characteristics of participants

**Table 1** summarises the demographics of the seventy-eight participants included in the analyses. Of these, 33 were resisters with Median [IQR]: IGRA, 0.04 [0-0.11] IU/mL and TST induration 0 [0-7] mm), 33 were with LTBI (IGRA, 4.59 [2.23-8.56] IU/mL and TST induration, 13 [10-15] mm) and 12 with discordant results (IGRA, 3.53 [1.79-8.17] IU/mL and TST induration 0 [0-0]mm) (**Supplementary Figure 1**). There were no statistically significant differences in the demographics and risk variables between the resisters and LTBI groups.

**Table 1:** Characteristics of participants included in the analyses and univariate analyses comparing resisters and LTBI groups.

		Resisters	LTBI	Discordant	P value (Overall) Resisters vs LTBI
Characteristics		TST-IGRA-	TST+IGRA+	TST=0mm IGRA≥1IU/mL	
**Sample size**		33	33	12	
**Sex**	Female	31 (94%)	25 (76%)	9 (75%)	0.086
	Male	2 (6%)	8 (24%)	3 (25%)	
**Age** **(years)**	Median (IQR)	42 (35.5 - 51)	42 (34.5 - 47)	44.5 (30.5 - 54)	0.665
**Ethnicity**	Black African	28 (85%)	29 (88%)	9 (75%)	0.592
	Mixed Race	3 (9%)	3 (9%)	3 (25%)	
	White	2 (6%)	0 (0%)	0 (0%)	
	Indian	0 (0%)	1 (3%)	0 (0%)	
**Duration of employment in TB setting**	5-10 years	27 (82%)	24 (73%)	9 (75%)	0.683
	10-15 years	4 (12%)	7 (21%)	2 (17%)	
	15+ years	2 (6%)	2 (6%)	1 (8%)	
**Other risk factors for infection**	Household exposure (last 20yrs)	6 (18%)	2 (6%)	1 (8%)	0.258
	Smoking (present or past)	3 (9%)	4 (12%)	2 (17%)	0.708

### 3.2 Concentrations of cytokines and chemokines in plasma were similar between groups

**Figure 1:**
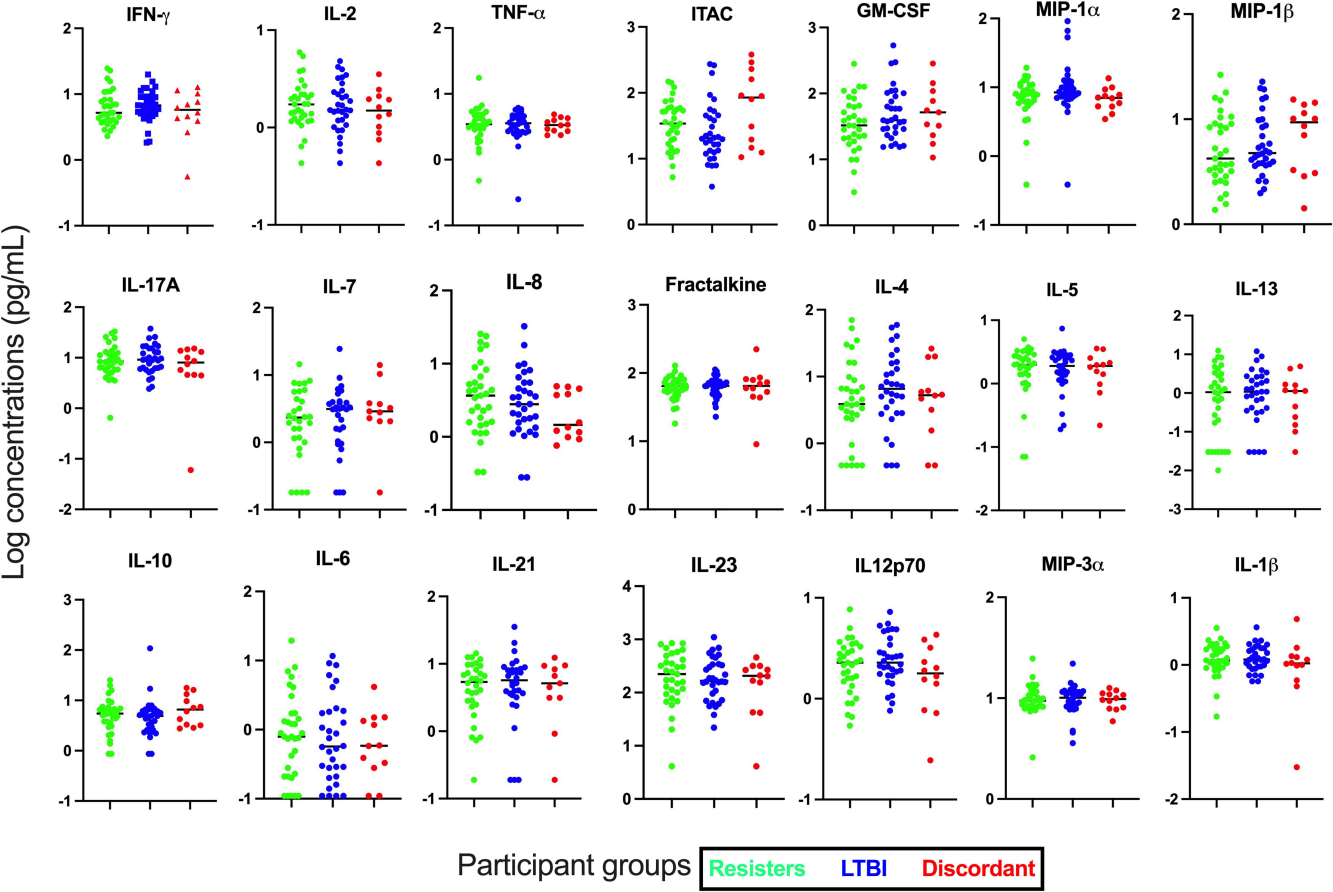
Measurement of cytokines and chemokines in plasma collected from fresh whole blood. Concentrations of 21 cytokines and chemokines were measured in plasma by Luminex. All graphs are in log scale.

First, we assessed the concentrations of cytokines and chemokines in plasma collected from whole blood without stimulation (**Figures 1**). Concentrations of the 21 cytokines and chemokines were determined and compared between the LTBI and resister groups. We observed that the concentrations of all these cytokines and chemokines were similar between the resisters and LTBI groups.

### 3.3 Mtb-specific Th1-associated cytokines and chemokines were significantly lower in resisters after stimulation

**Figure 2:**
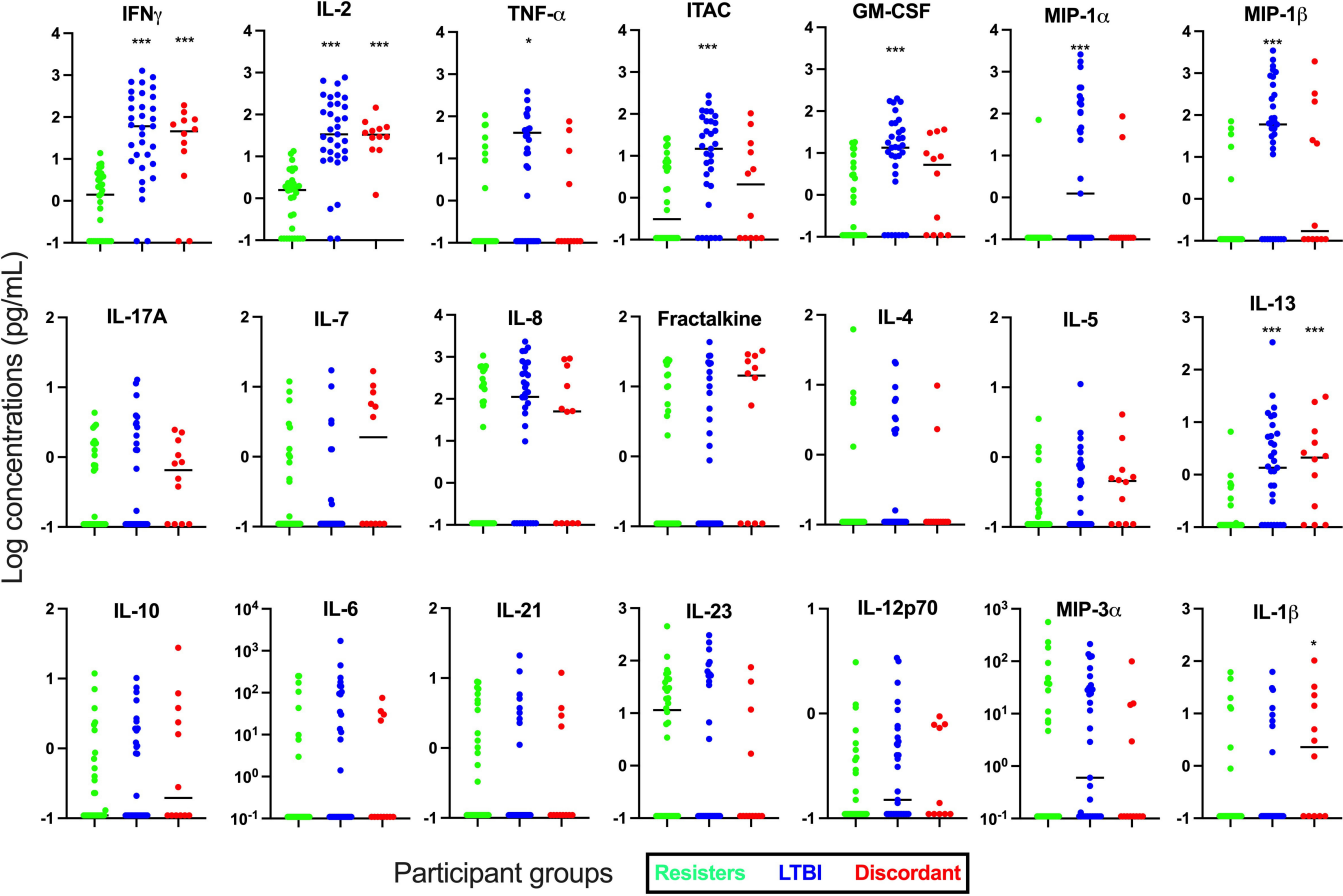
Measurement of cytokines and chemokines in supernatant collected from Quantiferon-TB Gold Plus tubes after incubation with Mtb-specific antigens (TB1) for 16-24 hours. Concentrations of 21 cytokines and chemokines were measured in supernatants by Luminex. Mann-Whitney test was used for statistical analyses and Bonferroni test was used to correct for multiple comparisons. *p value <0.05; ***p value <0.001 for LTBI vs Resisters or Discordant vs Resisters. All graphs are in log scale.

Next, we determined the concentration of these cytokines and chemokines in supernatants after stimulation with Mtb-specific antigens using the IGRA (TB1) tube, which stimulate mainly the CD4 responses (**Figure 2**). We observed that in addition to IFN-γ (median [IQR] concentrations in pg/mL; 1.47 [0-4.17] vs 60.31 [8.23-290.80], p<0.0001), cytokines and chemokines that are associated with mostly T cell immunity such as IL-2 (1.61 [0-3.62] vs 33.67 [9.74-177.10]; p<0.0001) and ITAC (0.51 [0-6.47] vs 14.72 [1.29-66.69]; p=0.006) were significantly lower in the resister group compared with the LTBI group. Other adaptive or innate immunity-related markers that were also significantly lower in the resister group compared with LTBI included TNF-α (0 [0-0.99] vs 6.07 [0-44.81], p=0.01), GM-CSF (0 [0-5.18] vs 13.46 [2.62-43.58]; p<0.0001), MIP-1α (0 [0-0] vs 1.24 [0-172.40]; p<0.0001) and MIP-1β (0 [0-0] vs 59.84 [0-415.30]; p<0.0001). We also observed significantly lower concentration of IL-13 (0 [0-0.19] vs 1.35 [0.02-5.76]; p<0.0001), which is traditionally a Th2 cytokine, in the resister group compared with LTBI group. There were no differences in the concentrations of innate cytokines/chemokines (IL-10, IL-1β, IL-23, MIP-3α, IL-6, IL-8, and IL-12p70), Th17-related cytokines (IL-17A and IL-21) and adaptive immunity-related cytokine/chemokine (IL-7 and Fractalkine) between the two groups. The concentrations of Th2-associated cytokines IL-4 and IL-5 were very low.

Next, we used principal component analyses (PCA) to evaluate the separation of the resisters from LTBI (**Supplementary Figure 2**) using cytokines from Mtb-antigen stimulation. When all analytes were plotted and when cytokines only were plotted, there was no clear separation between the two groups. When we evaluated chemokines only (MIP-1α, MIP-1β, MIP-3α, ITAC, Fractalkine and GM-CSF), there was a better separation between the resisters and LTBI groups, with some LTBI individuals classifying along with the resisters. PC1 contributed over 30% while PC2 contributed less than 20% of the variance.

### 3.4 Mtb-specific cytokines and chemokines in individuals with discordant results were comparable to those with LTBI

**Figure 3:**
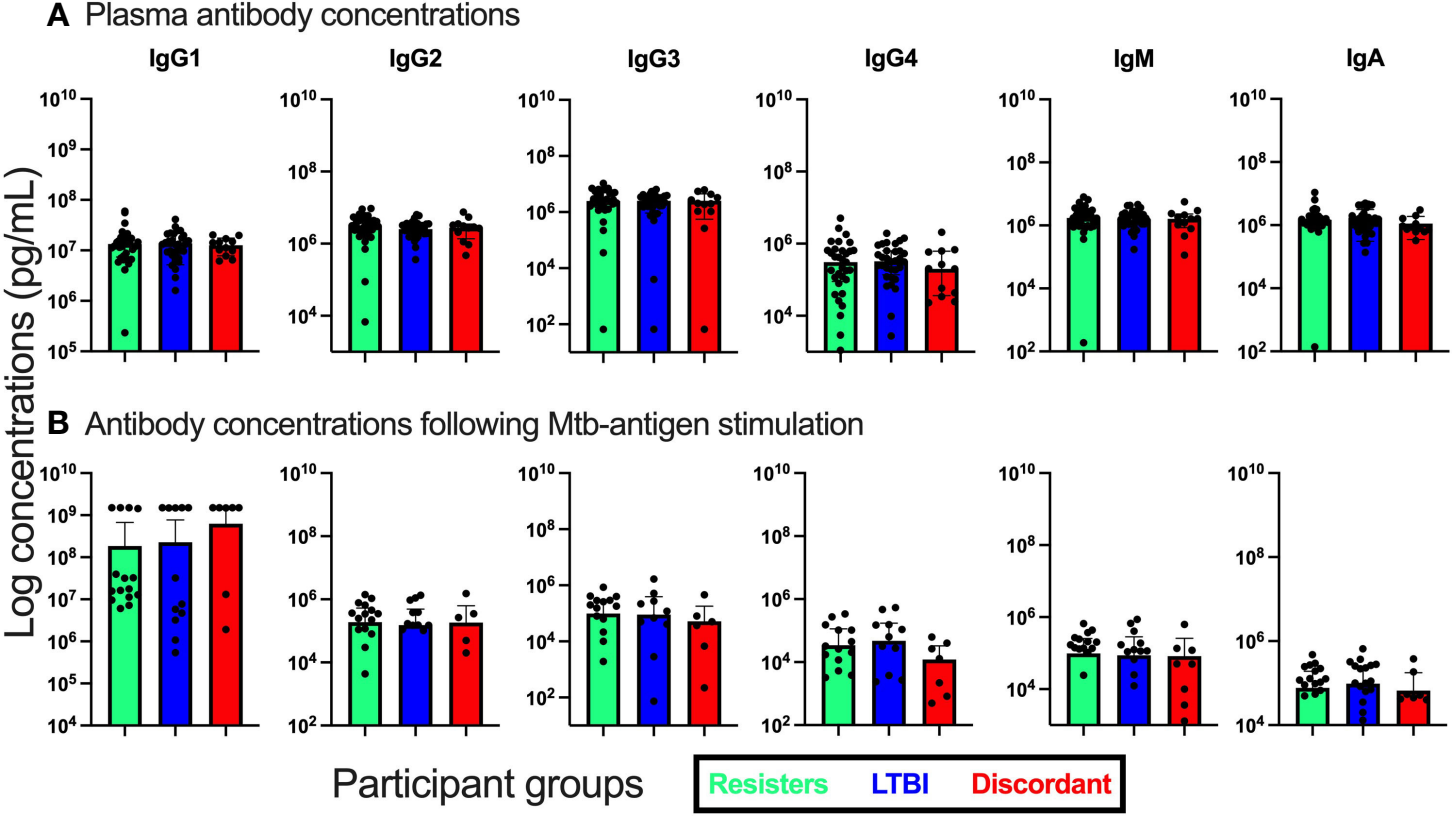
Measurement of antibody isotype concentrations in fresh plasma from unstimulated whole blood and supernatants from QuantiFERON-TB Gold Plus tubes after stimulation with Mtb-specific antigens for 16-24hours. **(A)**. Antibody concentrations in plasma. **(B)**. Antibody concentrations in supernatants.

We next compared concentrations of the cytokines between the discordant group with the other two groups, resisters and LTBI. We observed that concentrations of most of the cytokines and chemokines in the discordant group were similar to those in the LTBI group after Mtb antigen stimulation and followed the same trend as LTBI group when compared with the resister groups, even though most comparisons between discordant and resisters did not reach statistical significance (**Figure 3**). Concentrations of these cytokines and chemokines in plasma from the discordant group were similar to both the resister and LTBI groups.

### 3.5 IL-2 performed well as potential diagnostic marker for latent TB infection

Using our Luminex data from stimulated samples, we evaluated the diagnostic potential of the different cytokines for the diagnosis of latent TB infection. We performed Area-under-the-curve (AUC, ROC) analyses for each cytokine using the IFN-γ ELISA values from the IGRA test as reference. There was concordance between IFN-γ by ELISA and IFN-γ by Luminex (r=0.67, p<0.0001) (**Supplementary Figure 1D**). IL-2 (AUC [95% CI]; 0.8852 [0.7956 - 0.9749]; p<0.0001) performed well, even though still not as good as IFNγ measured by Luminex (0.8871 [0.8029 - 0.9712]; p<0.0001) in the diagnosis of LTBI. Other markers with high AUC values included GM-CSF (0.7764 [0.6614 - 0.8914]; p=0.0001), ITAC (0.7548 [0.6373 – 0.8723]; p=0.0004), and TNF-α (0.6625 [0.5302 - 0.7948]; p=0.02) (**Supplementary Figure 3**).

### 3.6 Total antibody concentrations in both supernatants and plasma were similar in resisters and LTBI

We also evaluated the concentrations of total IgA, IGM and IgG by subclass (IgG1, IgG2, IgG3, and IgG4) both in plasma and in supernatants after stimulation with Mtb specific antigens (**Figure 3**). First, in plasma, we observed that the concentrations of these antibody isotypes were similar between the three groups. We then assessed the concentrations in supernatants after stimulation and again observed no differences between the groups of participants.

## 4 Discussion

In this study, we investigated soluble immune markers in individuals with apparent resistance to MTB, recruited in a high incidence area. We show that demographic characteristics of participants with apparent resistance to Mtb infection were largely similar to those with LTBI. Plasma concentration of all cytokines evaluated were similar between the groups. Upon stimulation with Mtb antigens, concentrations of cytokines/chemokines mostly associated with a CD4 T cell or a Th1 response (IFN-γ, TNF-α, IL-2 and ITAC) as well as innate immunity-related cytokines/chemokines (GM-CSF, MIP-1α (CCL3) and MIP-1β (CCL4)) were significantly lower in the resister group compared with the LTBI group. Total concentrations of IgG subclasses, IgM, and IgA whether in plasma or supernatants after stimulation were similar between the groups. Given that a low to absent IFN-γ (a Th1 cytokine) response was used to define the resister group, we expected and found that other Th1 cytokines (IL-2 and TNF-α) were also significantly lower in resisters.

The importance of CD4 T cell immunity in TB is widely known, especially in people with HIV infection with low CD4 count who have an increased risk of developing active TB compared to those without HIV infection (10, 11). Mtb infection is diagnosed by memory/recall responses to Mtb-antigens by TST or IGRA assays. Individuals who do not show sign of infection despite exposure (resisters) may have an innate immune response that is able to clear the pathogen on its own through processes such as phagocytosis and autophagy, possibly with the help of effector cytokines produced early by some non-conventional T cell subsets. Non-conventional or donor-unrestricted T (DURT) cells such as mucosal-associated invariant T (MAIT) cells, gamma-delta T cells and invariant natural killer T (iNKT) cells that recognize non-protein antigens (12), may be involved in clearing the infection without the sensitization of the classical Th1 cells that will signify established infection. Alternatively, there may be alternate immune responses to Mtb infection in apparent resisters that do not involve the classical IFN-γ-related Th1 responses. A previous study of HHCs in Uganda reported that there were IFN-γ-independent CD4 T cell responses (expression of IL-2 with CD154/CD40L) and activation (measured by CD154/CD40L expression) to ESAT6/CFP10 in resisters that were comparable to LTBI (2). The expression of the combination of IL-2, TNF-α and IFN-γ on CD4 T cells was lower in resisters. The main difference between the HHCs study in Uganda and our study is that we measured soluble cytokines/chemokines in supernatant after stimulation, while they measured cytokine expression by CD4 T cells using flow cytometry after stimulation.

GM-CSF, ITAC, MIP-1α (CCL3) and MIP-1β (CCL4) are chemokines produced mainly by cells of the innate immune system such as macrophages but also by certain T cell subsets. These chemokines enable recruitment of T cells to the site of infection or homing to tissues where antigen presentation and immune activation occur (13). The secretion of GM-CSF, MIP-1α and MIP-1β during Mtb infection, together with IFN-γ, is essential for the differentiation of monocytes to macrophages and inhibition of Mtb growth in infected macrophages (14, 15). As IFN-γ may be essential for the secretion of these chemokines by innate immune cells (16), lower secretion of these markers in resisters may indicate the dependence on Th1 responses (mostly IFN-γ secretion) to induce these cytokines in macrophages and other cells of the innate immune system.

Despite lower concentrations of Th1-associated (IL-2) and innate/T cell (TNF-α, MIP-1β, MIP-1α, GM-CSF and ITAC) cytokines/chemokines in resisters compared to LTBI, many other innate immunity-related cytokines/chemokines (IL-10, IL-1β, IL-23, MIP-3α, IL-6, IL-8, and IL-12p70), Th17-related cytokines (IL-17A and IL-21) and adaptive immunity-related cytokines/chemokine (IL-7 and Fractalkine) were similar between resisters and LTBI groups. IL-1α, IL-1β and IL-18 are members of the IL-1 family of cytokines. These cytokines are centrally involved in the inflammasome activation and are produced by innate cells such as monocytes, macrophages and neutrophils (17–19). Deficiencies in these cytokines or their signaling pathways have been shown to result in increased lethality or susceptibility to Mtb infection in mice (20–22). IL-12, IL-23 and IL-6 belong to the same IL-6 superfamily of cytokines and are mainly produced by antigen presenting cells. These cytokines are essential for the development and differentiation of naïve T cells into different T cell subsets including Th1 and Th17. Deficiencies in the IL-12 pathway results in increased susceptibility to Mtb infection and TB disease (23). IL-17A and IL-23 form part of the Th17 immune responses and produced mainly by Th17 T cells but also by some lung-resident gamma-delta T cells early on during mycobacterial infection (24, 25). IL-17 plays a major role at the site of infection by promoting rapid accumulation of neutrophils and T cells in the lungs through induction of several chemokines, and when induced by IL-23, can result in an increase in Th1 immune responses relevant to the control of Mtb infection (26). CD4 T cells producing IL-17 in combination with TNF-α and IL-2 after BCG vaccination have been associated with control of Mtb infection (27). MIP-3α (CCL20), IL-8 (CXCL8) and Fractalkine (CX3CL1) are chemokines whose role in Mtb infection and disease have not been clearly defined but the levels of these markers or expression of their receptors (CCR6, CXCR1/2 and CX3CR1, respectively) have been shown to increase in Mtb infection or TB disease, regulating the Th1, Th17 immune responses as well as the recruitment of myeloid cells and production of reactive nitrogen intermediates (RNIs) (28–32). IL-10 as an anti-inflammatory or regulatory cytokine is produced by innate immune cells through the engagement of TLR as well as regulatory CD4 T cells (33). The major role of IL-10 is suppression of antigen presenting cell function including proinflammatory cytokine production induced by pathogenic infection in the periphery or site of infections including the granuloma, as well as inhibiting phagocytosis and microbial killing by nitrogen oxide intermediates induced by IFN-γ (34–36). Similar concentrations of these cytokines/chemokines in plasma between the resisters and LTBI suggest that resisters may have alternative physiological immune response compared with individuals with LTBI diagnosed by IGRA/TST.

As some high TB exposure settings including the location of this study, may rely only on TST induration to define Mtb infection among exposed individuals, we compared responses in resisters and LTBI to a group of participants with IGRA and TST extreme discordant results. Our observations that responses in individuals with no TST reactivity and a high IGRA value were more similar to LTBI than resisters show the importance of using both tests or IGRA to define Mtb infection or lack thereof. The lack of TST reactivity may not necessarily indicate the absence of infection.

In this study, we identified IL-2 as another potential diagnostic marker for latent TB infection. If confirmed in further studies, the use of IL-2 in combination with IFN-gamma may further improve diagnosis of latent TB infection. This finding has important clinical implications as it may improve the accuracy of TB diagnosis and lead to earlier treatment and better outcomes.

Studies have reported inconsistent results on the role of humoral immunity in Mtb infection and pathogenesis, but recent studies have demonstrated an important role for antibodies in controlling Mtb infection (37, 38). It has been shown that antibodies (IgG) derived from people with LTBI were more effective in preventing mycobacterial growth in macrophages *in vitro* compared with IgG derived from individuals with active TB (39). Similarly, IgA derived from individuals with LTBI and active TB inhibited Mtb growth in mucosal cell lines while IgG from the same individuals promoted Mtb infection in these cells (40). Serum antibodies against lipoarabinomannan (LAM) and arabinomannan derived from individuals immunized with BCG were able to promote phagocytosis and phagolysosome fusion leading to inhibition of intracellular growth of Mtb in macrophages (41). While there is promising data on the role of antibodies in preventing infection *in vitro*, more data is needed to directly show the importance of antibodies in preventing Mtb infection and or disease *in vivo* in humans. In the Uganda HHCs study, antibody responses to different Mtb antigens (PPD, Ag85, ESAT6/CFP10, HspX, GroES and LAM) were similar between the resisters and LTBI. This is in line with our current observations on total concentrations of IgG subclasses, IgM and IgA after Mtb-antigen stimulation. The difference between the HHCs study in Uganda and our study is that we measured concentrations of total antibodies, while they measured antibodies in response to Mtb-specific protein and lipid antigens.

The current study has a number of strengths and limitations. The major strength of the study is that we comprehensively screened for resistance to Mtb infection in high exposure environments using the two currently approved tests for diagnosis of Mtb infection. The limitations of the study are that we only evaluated 21 cytokines and chemokines as well as total concentrations of 6 antibody isotypes in plasma and in response to Mtb peptides and this may not reflect a complete picture of immune responses as these and other markers and cell subsets may show different responses to alternative Mtb-specific antigens or whole bacteria. We also did not have an additional control group of participants with low or no Mtb exposure to evaluate how their immune response compare to the other study groups. The resister phenotype was determined at a single timepoint and it is possible it might change overtime. A longitudinal study would be able to assess the changes. In addition, our sample size was relatively small, but we believe it was still adequate to show the differences between the study groups.

## 5 Conclusion

Despite lower Mtb-specific Th1-associated responses, resisters had similar concentrations of several other cytokines and chemokines and antibodies. This suggests an ability to mount non-IFN-γ immune responses that may be protective against Mtb-infection possibly by other T cell subsets. Further studies will evaluate cell-specific and additional soluble marker responses to live Mtb to assess if these are associated with resistance to Mtb infection.

## Data availability statement

The original contributions presented in the study are included in the article/**Supplementary Materials**, further inquiries can be directed to the corresponding author, Data is also available using the following link: https://zivahub.uct.ac.za/articles/dataset/Secretion_of_cytokines_chemokines_and_antibodies_in_individuals_with_resistance_to_Mycobacterium_tuberculosis_infection/22132736.

## Ethics statement

The studies involving human participants were reviewed and approved by University of Cape Town Faculty of Health Sciences Human Research Ethics Committee (HREC Ref 031/2019). The patients/participants provided their written informed consent to participate in this study.

## Author contributions

MS conceived the study, obtained funding, wrote all protocols and SOPs, processed and stored samples, performed laboratory experiments and analyses, supervised all aspects of the project and wrote the first draft of the paper. ABa, NM, and ABe processed and stored samples, performed the experiments and analyses, reviewed the manuscript. CS provided clinical and scientific inputs throughout the study, assisted with data analyses and reviewed the manuscript. RG and RD were involved in project management, recruitment of study participants and collection of biological samples and reviewed the manuscript. KW, DaL, and DeL provided critical scientific, clinical and data analysis input and reviewed the manuscript. PK advised and performed statistical analyses and reviewed the paper. GM was involved from project conception, provided critical scientific and clinical inputs, mentorship and reviewed the manuscript. All authors contributed to the article and approved the submitted version.

## Supplementary material

Supplementary Figure 1 IFN-γ and TST measurements. (A). IFNγ ELISA values measured by IGRA. (B). TST indurations. (C). Correlation between IFN-γ response to TB1 and TB2 antigens measured by ELISA. (D). Correlation between IFN-γ measured by ELISA in the IGRA assay (TB1) and IFN-γ measured by Luminex. For TST, zero values were replaced with 0.1; for IGRA, zero values were replaced with 0.001. For Luminex, zero values were replaced with 0.11 (lower limit of detection of the assay). Dotted lines in (A) and (B) indicated the cut-off values (0.35IU/mL for IGRA and 10mm for TST). All graphs are in log scale.

Supplementary Figure 2 Principal components analysis was used to compare LTBI with resisters for all markers analyzed (A), cytokines only (B) or chemokines only (C). Participants are represented by dots and colored by outcome (green for LTBI and orange for resisters). The two axes represent principal components 1 (PC1/Dim1 on the x-axis) and 2 (PC2/Dim2 on the y-axis), and their contribution to the total data variance is shown as a percentage.

Supplementary Figure 3 Assessing the utility of cytokines measured after stimulation with Mtb-specific antigens as diagnostic markers for latent TB infection. (A). Area-under-the curve analyses for selected cytokines were performed using receiver operator characteristics in R version 4.0.4. (B). AUC values for all cytokines measured.
