# Supplementary figures and images for: Mycobacterial-specific secretion of cytokines and chemokines in healthcare workers with apparent resistance to infection with Mycobacterium tuberculosis
Source: Front Immunol. 2023 May 18;14:1176615. doi: 10.3389/fimmu.2023.1176615 (PMC10233115; doi:10.3389/fimmu.2023.1176615)

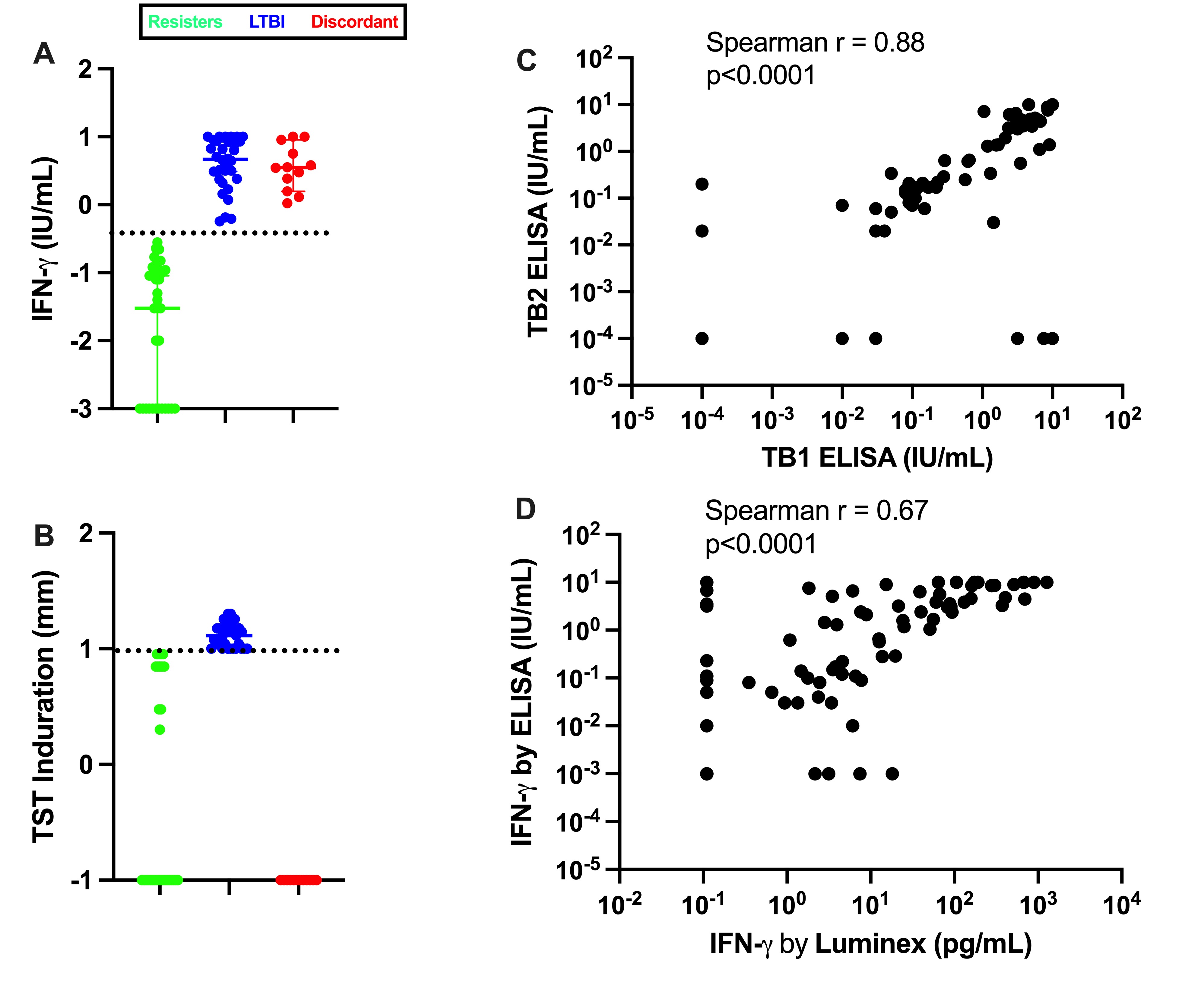

Supplement: Supplementary Figure 1 — IFN-γ and TST measurements. (A). IFNγ ELISA values measured by IGRA. (B). TST indurations. (C). Correlation between IFN-γ response to TB1 and TB2 antigens measured by ELISA. (D). Correlation between IFN-γ measured by ELISA in the IGRA assay (TB1) and IFN-γ measured by Luminex. For TST, zero values were replaced with 0.1; for IGRA, zero values were replaced with 0.001. For Luminex, zero values were replaced with 0.11 (lower limit of detection of the assay). Dotted lines in (A) and (B) indicated the cut-off values (0.35IU/mL for IGRA and 10mm for TST). All graphs are in log scale. [file Image_1.jpeg]

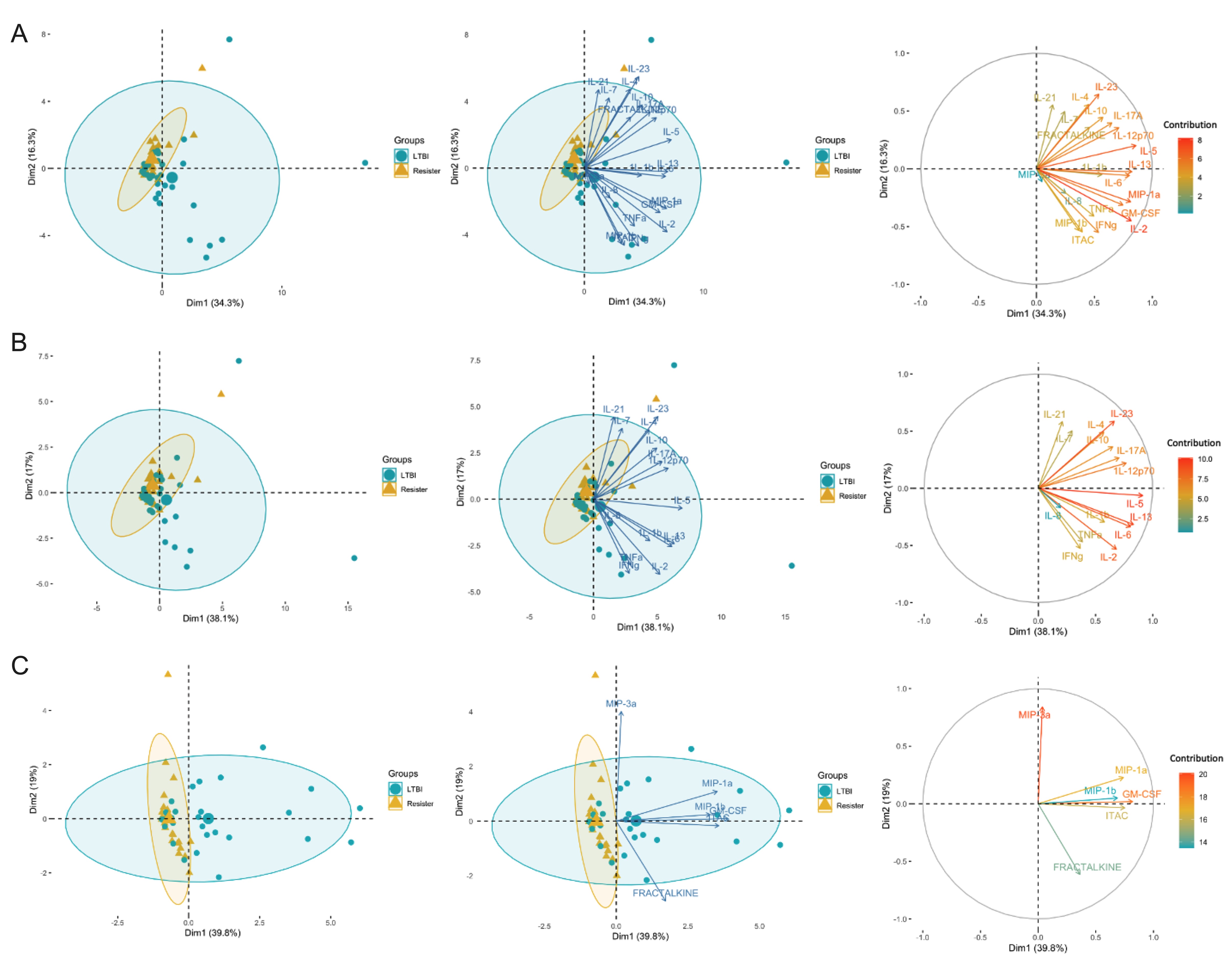

Supplement: Supplementary Figure 2 — Principal components analysis was used to compare LTBI with resisters for all markers analyzed (A), cytokines only (B) or chemokines only (C). Participants are represented by dots and colored by outcome (green for LTBI and orange for resisters). The two axes represent principal components 1 (PC1/Dim1 on the x-axis) and 2 (PC2/Dim2 on the y-axis), and their contribution to the total data variance is shown as a percentage. [file Image_2.jpeg]

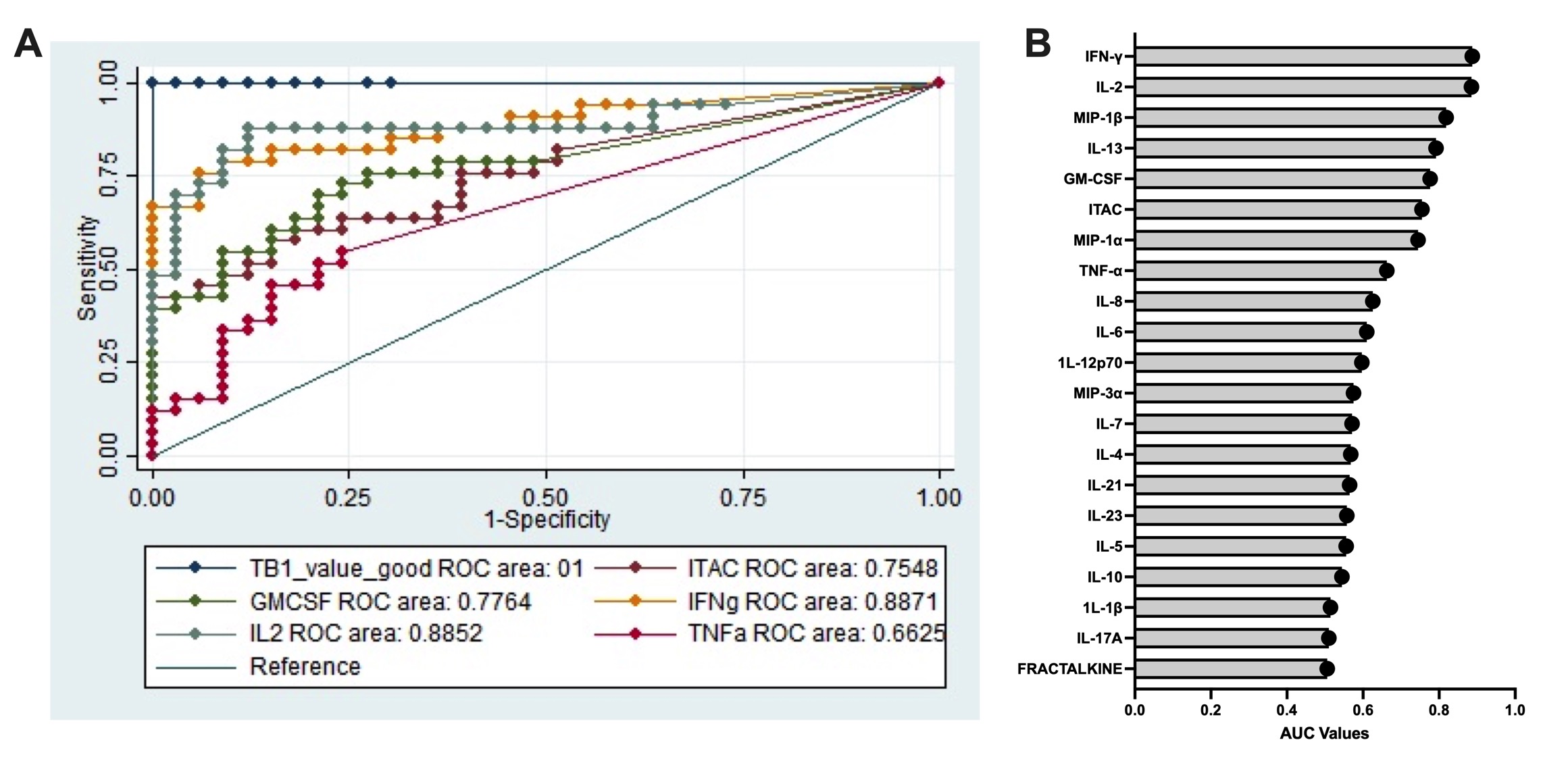

Supplement: Supplementary Figure 3 — Assessing the utility of cytokines measured after stimulation with Mtb-specific antigens as diagnostic markers for latent TB infection. (A). Area-under-the curve analyses for selected cytokines were performed using receiver operator characteristics in R version 4.0.4. (B). AUC values for all cytokines measured. [file Image_3.jpeg]
